# Porous Carbon Boosted Non-Enzymatic Glutamate Detection with Ultra-High Sensitivity in Broad Range Using Cu Ions

**DOI:** 10.3390/nano12121987

**Published:** 2022-06-09

**Authors:** Yifei Ma, Jiemin Han, Zhaomin Tong, Jieling Qin, Mei Wang, Jonghwan Suhr, Jaedo Nam, Liantuan Xiao, Suotang Jia, Xuyuan Chen

**Affiliations:** 1 State Key Laboratory of Quantum Optics and Quantum Optics Devices, Institute of Laser Spectroscopy, Collaborative Innovation Center of Extreme Optics, Shanxi University, Taiyuan 030006, China; mayifei@sxu.edu.cn (Y.M.); jiemin.han@foxmail.com (J.H.); zhaomin.tong@sxu.edu.cn (Z.T.); xlt@sxu.edu.cn (L.X.); tjia@sxu.edu.cn (S.J.); xuyuan.chen@usn.no (X.C.); 2 Tongji University Cancer Center, Shanghai Tenth People’s Hospital of Tongji University, School of Medicine, Tongji University, Shanghai 200092, China; 3 Department of Polymer Science and Engineering, Sungkyunkwan University, Suwon 16419, Korea; suhr@skku.edu (J.S.); jdnam@skku.edu (J.N.); 4 School of Mechanical Engineering, Sungkyunkwan University, Suwon 16419, Korea; 5 Department of Micro- and Nanosystem Technology, Faculty of Technology and Maritime Sciences, University College of Southeast Norway, 3184 Borre, Norway

**Keywords:** carbon foam electrode, copper ion chelation, glutamate detection, high sensitivity

## Abstract

A non-enzymatic electrochemical sensor, based on the electrode of a chitosan-derived carbon foam, has been successfully developed for the detection of glutamate. Attributed to the chelation of Cu ions and glutamate molecules, the glutamate could be detected in an amperometric way by means of the redox reactions of chelation compounds, which outperform the traditional enzymatic sensors. Moreover, due to the large electroactive surface area and effective electron transportation of the porous carbon foam, a remarkable electrochemical sensitivity up to 1.9 × 10^4^ μA/mM∙cm^2^ and a broad-spectrum detection range from nM to mM scale have been achieved, which is two-orders of magnitude higher and one magnitude broader than the best reported values thus far. Furthermore, our reported glutamate detection system also demonstrates a desirable anti-interference ability as well as a durable stability. The experimental revelations show that the Cu ions chelation-assisted electrochemical sensor with carbon foam electrode has significant potential for an easy fabricating, enzyme-free, broad-spectrum, sensitive, anti-interfering, and stable glutamate-sensing platform.

## 1. Introduction

Huntington’s disease (HD) is one of the most prevalent neurodegenerative diseases (NDs), of which the symptoms typically begin in elderly age [1,2]. Similar to other NDs, the symptoms of HD are generally mild at the start yet become worse over time and interfere with daily life [3]. Several researchers have realized that the presence of glutamate in the cerebral cortex is one of the key points for intracellular signal pathways, and the concentration change of glutamate is possibly related to HD [1,4,5]. In addition, glutamate is also an important biomarker for other diseases, such as musculoskeletal pain [6], tumor cells [7], and Alzheimer’s disease [8]. Hence, the detection of glutamate can be applied in clinical diagnoses as well as symptom monitoring during the treatments of these diseases [9,10]. The concentrations of glutamate in plasma, serum, cerebral spinal fluid, urine, whole blood and saliva are in the range of 5–100 μM, 97.4 ± 13.2 μM, 0.5–2 μM, 8.5 (3.3–18.4) μM mM^−1^ creatinine, 150–300 μM, and 0.232 ± 0.177 μM, respectively [11]. Therefore, the broader the detection range of glutamate is, the better, and the limit of detection of the non-enzymatic glutamate sensors should be at least lower than 0.05 μM.

Recent studies have reported a number of electrochemical biosensors for the detection of glutamate [12,13,14]. Although enzymatic glutamate biosensors such as electrochemical sensors using L-glutamate oxidase (GluO_x_) have demonstrated their capability for glutamate detection, their low sensitivity is problematic due to indirect electron transfer [15]. Moreover, a few other shortcomings of enzymatic detection techniques also need to be overcome, including the complicated enzyme purification procedures, high fabrication costs, instability due to enzyme denaturation, and a narrow detection range. In addition, in terms of the mechanism of glutamate detection in an electrochemical strategy, catalyzing oxidative deamination of glutamate by the enzymes, and the redox reaction of glutamate to oxoglutarate, which is ascribed from the catalytic effect of multivalent cationic metal ions, are the main methods used thus far [16,17]. Unfortunately, the current strategies can only offer a sensitivity of up to ~10^2^ μA/mM∙cm^2^ and a detection linear range in the μM scale, which cannot fulfill the detection requirements. In this regard, undoubtedly, developing a broad-spectrum and highly sensitive glutamate-sensing system is extremely important.

When addressing such formidable challenges of enzymatic detection, researchers have made great efforts to exploit non-enzymatic sensors, which are called the fourth generation of electrochemical glutamate sensors. Of the various factors previously highlighted for establishing a high-performance non-enzymatic glutamate sensor, nanostructured materials as sensing platforms hold an effective strategy for high sensitivity and broad detective concentration ranges which ascribe from their large electrochemically active surface area, as well as a desirable anti-interference, short response time and impressive stability. Therefore, developing nanostructured materials that boosted non-enzymatic glutamate sensors is an irresistible trend that could improve the stability and decrease the cost of sensors. For example, Razeeb et al. firstly developed a non-enzymatic Pt/Ni nanowire array electrode to detect glutamate in 2012 [16]. Disappointingly, despite the complicated and costly synthesis process of the precisely structured nanowire, the sensitivity and linear range of detection were far removed from expectations. Since then, even though the non-enzymatic glutamate sensors have been developed for ten years, there are still a limited number of works of literature published [18,19,20,21,22,23]. Islam et al. reported RuO_2_-doped ZnO nanoparticles based on a non-enzymatic glutamate sensor, which reports a high sensitivity of up to 9.6 × 10^−5^ μA/mM∙cm^2^ and the lowest detection limit of 0.0001 μM [22]. However, the highest detection limit of this sensor is only 10 μM, which cannot fulfill the detection requirement in many clinical environments, such as plasma, serum, and whole blood.

Owing to its affinitive chelation with Cu ions, glutamate has been employed as a chelation agent to enhance the electrodeposition of copper and prevent the precipitation of copper oxide [24]. This motivated us to develop a Cu ions chelation-assisted system for high-performance glutamate sensing. Along with our recent advance in the synthesis technique of porous carbon foams, we were able to directly detect the concentration of glutamate in an amperometric way. Contributing to the large electroactive surface area and effective electron transportation of the chitosan-derived carbon foam electrode, a high electrochemical sensitivity and a broad-spectrum detection range can be achieved. This study describes the new strategy of a facile and non-enzymatic detection of glutamate, assisted by chelating with Cu ions, and to the best of our knowledge, it reports the highest sensitivity and broadest detection range thus far.

## 2. Experimental

### 2.1. Chemicals and Apparatus

L-glutamic acid monosodium salt monohydrate (glutamate, ≥98%), copper chloride (CuCl_2_, 97%), ascorbic acid (AA), uric acid (UA), dopamine hydrochloride (DA), glucose, 3,4-Dihydroxyphenylacetic acid (DOPAC), chitosan (medium molecule weight), and acetic acid were purchased from Sigma-Aldrich (USA). Phosphate-buffered saline (10 mM of PBS, pH = 7.4) was prepared from NaCl, KCl, Na_2_HPO_4_, and KH_2_PO_4_. All chemicals were commercially available at analytical grade and were used without further purification.

### 2.2. Preparation of Electrode Based on a Chitosan-Derived Carbon Foam

The chitosan-derived carbon foam was synthesized from a chitosan foam, which was prepared through a temperature-controlled freeze-casting process [25]. In brief, chitosan powders were dissolved into a 0.3 M acetic acid solution at a chitosan concentration of 10 mg/mL. Subsequently, the solution was frozen at −20 °C and lyophilized in a freeze-dryer at −80 °C for 48 h. Afterwards, the resultant chitosan foam was annealed at 900 °C for 2 h to obtain a cylindrical chitosan-derived carbon foam. The carbon foam was cut with a thickness of 1 mm and attached to a gold plate (as a current collector) with a conductive carbon tape.

### 2.3. Characterizations

The morphology of the chitosan-derived carbon foam was examined with field emission scanning electron microscopy (FE-SEM, HITACHI, SU8010, Tokyo, Japan). The pyrolysis information was obtained from thermal gravimetric analysis (TGA, Mettler Toledo, TGA 2, Zurich, Switzerland). Focused monochromatized Al Kα radiation (hν = 1486.6 eV) was utilized for the X-ray photoelectron spectroscopy (XPS, ESCALAB, Thermo-Scientific, Brno, Czech). All electrochemical measurements were performed using a VSP potentiostat (Princeton Applied Research, Oak Ridge, TN, USA) at room temperature. A conventional three-electrode system consists of the carbon foam electrode, a platinum plate and Ag/AgCl (saturated KCl solution) as the working, counter and reference electrodes, respectively. The electrochemical performance of the carbon foam electrode on glutamate was studied via cyclic voltammetry (CV) between −0.55 and 0.65 V at a scan rate of 100 mV/s in 10 mM PBS containing 2, 4, and 6 mM CuCl_2_, respectively. The amperometric responses were operated by chronoamperometry (CA) in 10 mM PBS containing 4 mM CuCl_2_ at the excited potentials of 0.03 V and 0.31 V, obtained from the previous CV.

## 3. Results and Discussion

### 3.1. Chitosan-Derived Carbon Foam Electrode

**Figure 1:**
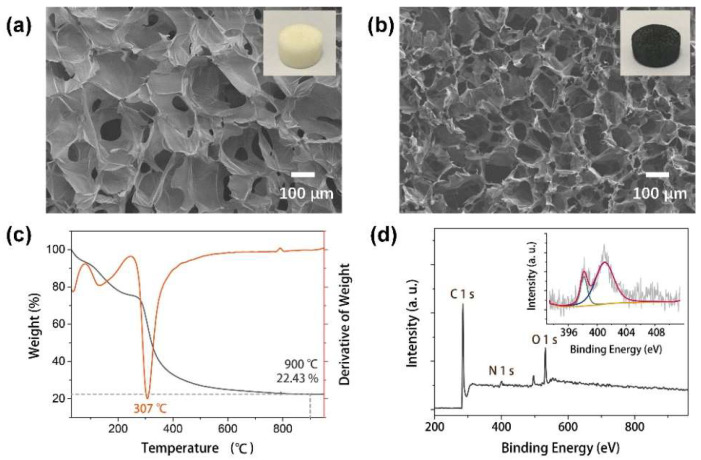
(**a**) SEM image of chitosan foam; (**b**) SEM image of chitosan-derived carbon foam; (**c**) TGA thermogram of weight loss and its derivative of chitosan foam; (**d**) X-ray photoelectron spectroscopy (XPS) wide scan spectrum and deconvoluted spectra of N 1 s (inset) of carbon foam.

The chitosan-derived carbon foam was synthesized from a precursor of a chitosan foam made through a freeze-casting process, as illustrated in Figure S1 from Supplementary Materials [25]. The chitosan foam exhibited a porous cellular structure, with the chitosan chains connected with each other, as shown in Figure 1a. After the subsequent pyrolysis process to prepare the carbon foam, the cellular structure kept well while the pore size obviously shrinks (Figure 1b). The change in the pore size was attributed to the weight loss of the chitosan, where the weight after pyrolysis at 900 °C only remained at 22.43% (Figure 1c). The decomposition of chitosan happened at the temperature of ~307 °C and the weight loss occurred steadily at 900 °C, where the porous carbon foam was well synthesized. XPS was checked to precisely demonstrate the N-doping in the carbon foam and chitosan foam, as shown in Figure 1d and Figure S2 from Supplementary Materials. The content of nitrogen in the carbon foam was 5.65%, which was derived from the nitrogen groups in chitosan foam and confirmed from the N 1 s peak in the wide scan of XPS spectra. The deconvoluted N 1 s peak of the N-doped carbon foam shows two distinguished peaks at 398.2 and 401.1 eV, which are attributed to pyridinic N and graphitic N, respectively [26,27,28], indicating the successful N-doping in the carbon foam during the pyrolysis process.

### 3.2. The Electrochemical Characterization

**Figure 2:**
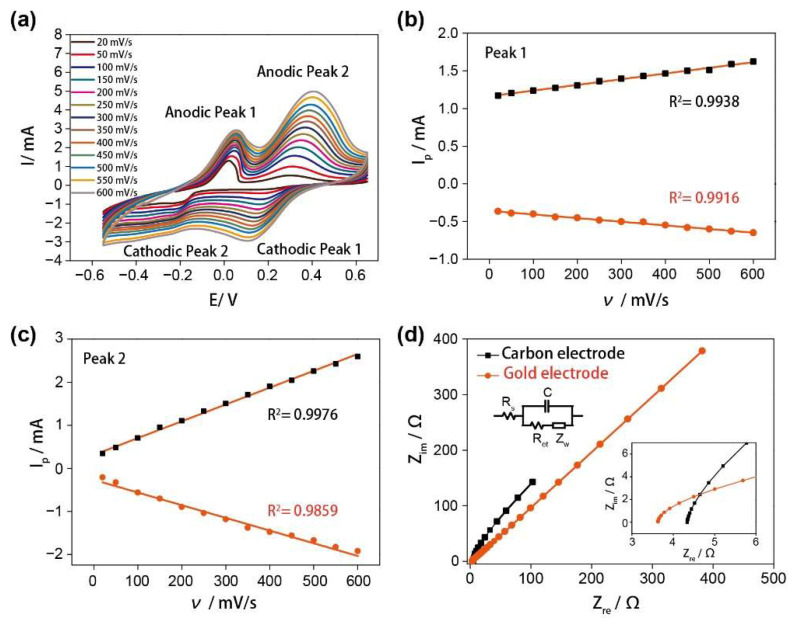
(**a**) Cyclic voltammograms for 4 mM CuCl_2_ chelation agent in 100 μM glutamate in PBS at scan rates of 20, 50, 100, 150, 200, 250, 300, 350, 400, 450, 500, 550, and 600 mV/s; (**b**) currents of redox peak 1 obtained from (**a**) as functions of the scan rates; (**c**) currents of redox peak 2 obtained from (**a**) as functions of the scan rates; (**d**) Nyquist plots of carbon-based electrode and flat gold electrode in 10 mM PBS containing 100 μM glutamate and 4 mM CuCl_2_ solution.

Porous nanocarbon materials, such as carbon nanotubes, have been widely used as biosensor electrodes for the detection of water-soluble species [29]. In our study, the highly porous chitosan-derived carbon foam was utilized as an electrode for glutamate detection. Figure 2a showed the cyclic voltammograms of the electrode in 10 mM PBS containing 100 μM of glutamate and 4 mM CuCl_2_ at different potential sweep rates in a wide range of 20–600 mV/s. The dependence of the anodic and cathodic peak currents of glutamate on the scan rates (ν) was depicted in Figure 2b,c. As shown in these figures, the currents of both the oxidation and reduction peaks increased with the increasing scan rates and the peak-to-peak separations also increased simultaneously. The linear regression equations were obtained as follows:

Peak 1: I_pa_ = 7.55 × 10^−4^ ν + 1.1625, R^2^ = 0.9938

I_pc_ = −4.84 × 10^−4^ ν − 0.3570, R^2^ = 0.9916

Peak 2: I_pa_ = 0.0039 ν + 0.3239, R^2^ = 0.9976

I_pc_ = −0.0029 ν − 0.2542, R^2^ = 0.9859

The perfect linear relationship between the current and scan rates indicates a deposition-controlled process (also called the surface-controlled process), which is ideal for glutamate detection [30,31,32].

The electrochemical properties of our carbon foam electrode and a gold electrode were evaluated by electrochemical impedance spectroscopy (EIS) in Figure 2d. The electroactive surface areas of these two electrodes were estimated from EIS data and presented in Table S1 [33,34]. The R_s_, Z_w_, R_et_, and C in the equivalent circuit represent the solution resistance, the Warburg diffusion resistance, the electron-transfer resistance, and the double-layer capacitance, respectively [35]. Moreover, the electrochemically active specific surface area (S_A_) can be calculated from the specific capacitance of the electrochemical double-layer by means of the relationship S_A_ = C/C_d_, where C_d_ is a constant value of 20 μF/cm^2^ [36]. As shown in Table S1, the calculated S_A_ of the carbon-based electrode is 11.66 cm^2^/g, which is about 12 times higher than the commonly used gold electrode (0.98 cm^2^/g). It indicates that the chitosan-derived carbon foam electrode can effectively provide a large active surface area, and its intrinsic porous structure can enhance the mass transport of glutamate and decrease the diffusion pathway to reach excellent electrochemical performance for glutamate detection.

### 3.3. The Sensing Performances

**Figure 3:**
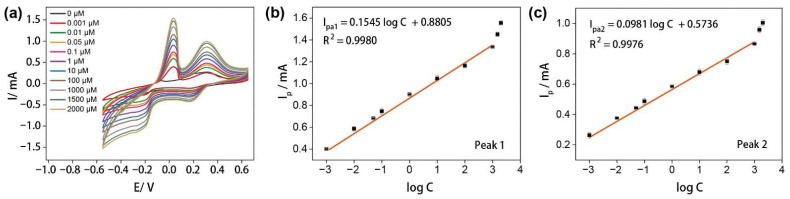
(**a**) Cyclic voltammograms of carbon-based electrode for the detection of the different concentrations of glutamates (0, 0.001, 0.01, 0.05, 0.1, 1, 10, 100, 1000, 1500, and 2000 μM) in 10 mM PBS containing 4 mM CuCl_2_; (**b**) calibration curve of I_p_ vs. log C of anodic peak 1; (**c**) calibration curve of I_p_ vs. log C of anodic peak 2.

Figure 3a showed the CV response of the carbon-based electrode when detecting glutamate in 10 mM PBS (pH = 7.4) containing 4 mM CuCl_2_ at the applied potentials between −0.55 and 0.65 V with a scan rate of 100 mV/s. It is worth noting that, without the existence of glutamate, the CV plot of 4 mM CuCl_2_ presents no obvious peak compared with that of the glutamate solutions with different concentrations. While at the appearance of glutamate, the glutamate would chelate with Cu^2+^, thereby forming [CuGlu_2_]^2-^ [24,37]. Hence, the redox peaks are ascribed from the electro-oxidation and electro-reduction of [CuGlu_2_]^2-^. As shown in Figure 3b,c, the currents are proportional to the logarithmic concentration of glutamate over the range of 0.001 to 1000 μM for peaks 1 and 2 at the potential of 0.03 and 0.31 V, respectively. The linear regression equations of the anodic peaks are I_pa1_ = 0.1545 log C + 0.8805, R^2^ = 0.9980 and I_pa2_ = 0.0981 log C + 0.5736, R^2^ = 0.9976, with the relative standard deviation (RSD) of 3.15% and 2.32%, respectively. The anodic peaks represent the oxidation of the copper chelate compounds, while the cathodic peaks around 0.17 V and −0.28 V are assigned to the reduction of the chelate compounds.

For confirming the good performance of the carbon foam electrode, the gold plate was undertaken as the electrode for glutamate detection, as shown in Figure S3 (from Supplementary Materials). It can be observed that the current obtained from the porous carbon electrode is 10 times higher than that of using a gold plate electrode at the glutamate concentration of 1 mM, and the glutamate can only be detected under the high glutamate concentration of 0.5–2 mM. The good performance of the carbon electrode is ascribed to the high specific surface area of the carbon foam, which can supply plenty of reaction sites and increase the electrochemical signal. Moreover, the chitosan-derived carbon foams possessed the intrinsic N-doped nature (Figure 1d) [38,39,40], which can also improve the hydrophilicity of carbon foam electrode and the affinity of the electrode and glutamate, resulting in a broad glutamate detection range using the carbon foam electrode. In addition, Figure S4 (from Supplementary Materials) exhibits the CV curve of the 4 mM CuCl_2_ aqueous solution using the carbon foam electrode and Figure S5 (from Supplementary Materials) shows the detection of glutamate without the CuCl_2_. No obvious redox peak can be observed in the CV curve of sole Cu^2+^ and the currents of the anodic peak exhibit no obvious difference with the increasing glutamate concentration without Cu^2+^, demonstrating the effectiveness of the Cu^2+^ chelation-assisted detection system.

In order to ensure the effectiveness of the detection method at 4 mM CuCl_2_, glutamate detection was also conducted at 2 and 6 mM CuCl_2_, as shown in Figure S6 (from Supplementary Materials). Under the condition of 2 mM CuCl_2_, only peak 2 existed in the plots at various concentrations of glutamate, while both peaks 1 and 2 appeared when the concentration of CuCl_2_ was 6 mM. The currents in peak 1 show a good linear relation with the log C (R^2^ = 0.9926) for the detection of glutamate from 0.01 to 1000 μM, however, the R^2^ at 4 mM CuCl_2_ is higher than that in 6 mM CuCl_2_, indicating a better performance for the glutamate detection than 2 and 6 mM CuCl_2_.

### 3.4. The Sensing Mechanisms

**Figure 4:**
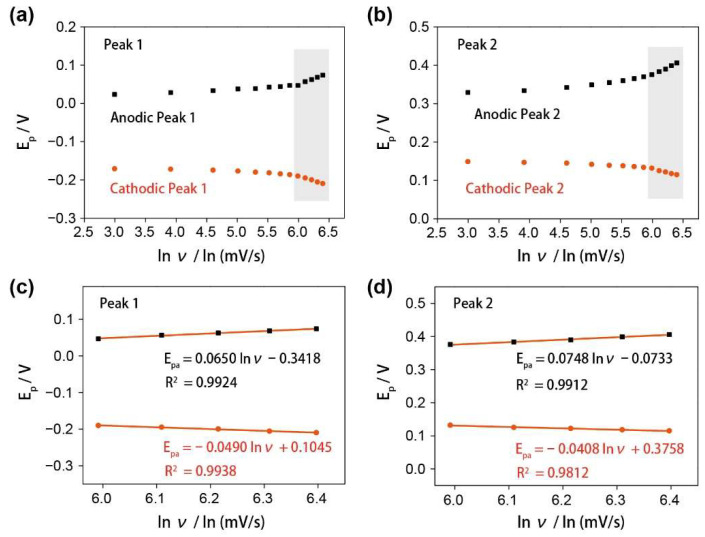
(**a**) Variations of E_p_ vs. ln ν of the redox peak 1; (**b**) variations of E_p_ vs. ln ν of the redox peak 2. (**c**) The calibration curves of E_p_ vs. ln ν of the redox peak 1 when ν > 400 mV/s. (**d**) The calibration curves of E_p_ vs. ln ν of the redox peak 2 when ν > 400 mV/s.

In order to understand the mechanism of the glutamate detection resulting from the complex formation of Cu ions, the electron-transfer mechanism during the electrochemical reactions was investigated. A good linear relationship between the potentials (E_p_) of redox peaks and the logarithm of the scan rates (ln ν) were plotted and shown in Figure 4a,b. Laviron derived general expressions for the linear potential scan voltammetric response are as follows [41,42]:

E_pa_ = E_0_ + A ln ν (1)

E_pc_ = E_0_ + B ln ν (2)

where the A = RT/(1−α)nF, and the B = RT/αnF. E_pa_ and E_pc_ are the anodic and cathodic peak potentials, respectively, and the α, K_s_, n and ν are the electron-transfer coefficient, the apparent charge-transfer rate constant, number of electron transfer, and potential sweep rate, respectively. From these expressions, it is possible to determine the α by measuring the variation of the peak potentials with scan rates and the n can be determined for the electron-transfer number between the electrode and the surface-deposited layer by measuring the E_p_ values (R = 8.314 J/K∙mol, T = 298 K, F = 96,485 C/mol). Plots of the E_pa_ and E_pc_ as functions of the ln ν yield two straight lines with slopes equal to RT/(1 − α)nF and RT/αnF for the anodic and cathodic peaks, respectively. Figure 4c shows the plot of E_p_ versus ln ν with slopes equal to 0.0650 and −0.0490 for anodic and cathodic peaks 1, respectively. Using the slopes of plots, the value of α was specified as 0.57 and the electron-transfer number was 1 (0.9194). Figure 4d shows the plot of E_p_ versus ln ν with slopes equal to 0.0748 and −0.04083 for anodic and cathodic Peaks 2, respectively, thereby the value of α was specified as 0.648 and the electron-transfer number is 1 (0.9722). Hence, all the electron transfers of these two redox peaks are both 1.

During the cyclic voltammetric process, dark brown copper appears on the carbon foam electrode at the end of the cathodic process. According to this phenomenon and the calculated electron-transfer number, the reaction mechanism could be speculated as follows:

Cathodic peak 1: [Cu^II^(Glu)_2_]^2−^ + e^−^ → [Cu^I^(Glu)_x_]^n^ (3)

Cathodic peak 2: [Cu^I^(Glu)_x_]^n^ + e^−^ → Cu^0^ + Glu^2−^ (4)

Anodic peak 1: Cu^0^ + Glu^2−^ − e^−^ → [Cu^I^(Glu)_x_]^n^ (5)

Anodic peak 2: [Cu^I^(Glu)_x_]^n^ − e^−^ → [Cu^II^(Glu)_2_]^2−^ (6)

where the [Cu^I^(Glu)_x_]^n^ stands for the complex formed between Glu^2−^ and Cu^+^ [24,43]. Under the appearance of copper ions, chelation compounds of [Cu^II^(Glu)_2_]^2−^ are formed [44,45], and subsequently, the intermediate [Cu^I^(Glu)_x_]^n^ and final product Cu^0^ are synthesized on the carbon foam electrode after the cathodic peak 1 and peak 2, respectively [24,46]. Therefore, in the anodic process afterwards, glutamate interacts with Cu^I^ or Cu^II^ to form the chelation compounds, in which the glutamate can be detected in an amperometric way due to the redox reactions of Cu.

**Figure 5:**
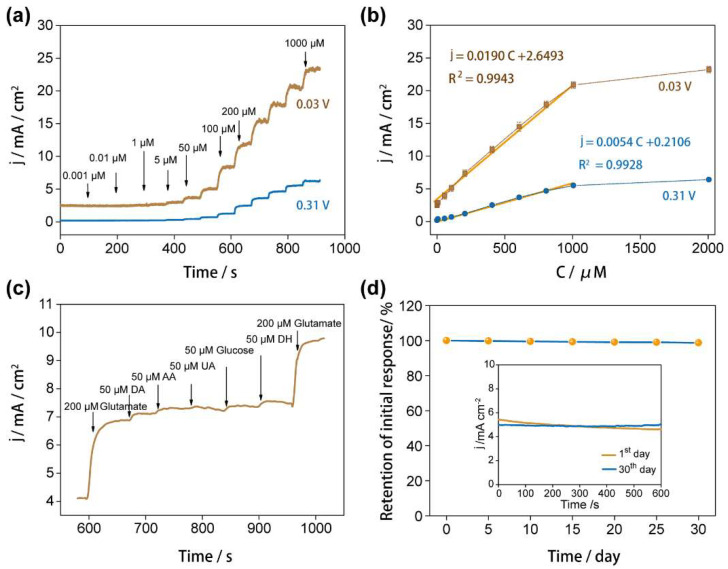
(**a**) Amperometric responses of carbon-based electrode with stepwise addition of glutamate stock solution at +0.03 V and +0.31 V; (**b**) plots of the response currents from (**a**) against the concentration of glutamate; (**c**) amperometric response of carbon foam electrode in successive addition of 200 μM glutamate, 50 μM of different interference species (3,4-Dihydroxyphenylacetic acid (DA), ascorbic acid (AA), uric acid (UA), glucose, and dopamine hydrochloride (DH) in 10 mM PBS containing 4 mM CuCl_2_ at 0.03 V; (**d**) long-term stability of the carbon foam electrode measured in 30 days.

The amperometric sensing performances of glutamate were carried out under the oxidation potentials of +0.03 V and +0.31 V, respectively. Figure 5a showed typical amperometric response curves of the successive addition of 0.001, 0.01, 1, 5, 50, 100, 200, and 1000 μM of glutamate in 10 mM PBS containing 4 mM CuCl_2_ for the carbon foam electrode. The current response increased directly after adding the glutamate and achieved a steady-state within 10 s, suggesting the fast rate of electron transfer between glutamate and our proposed electrode. In the calibration curves (Figure 5b), the carbon-based electrode provides a linear range of glutamate from 0.001 to 1000 μM. The linear regression equations are [31,47]: j (mA/cm^2^) = 0.0190 C (μM) + 2.6493, R^2^ = 0.9943 for 0.03 V; and j (mA/cm^2^) = 0.0054 C (μM) + 0.2106, R^2^ = 0.9928 for 0.31 V on carbon foam electrode. Compared with the other reported electrochemical sensors for the detection of glutamate in Table 1, the carbon-based sensor in this study exhibits the highest sensitivity (1.9 × 10^4^ μA/mM∙cm^2^) as well as the comparable detection limit (0.001 μM) and linear range (0.001–1000 μM).

Selective electrochemical detection of glutamate is a challenging task because the oxidizable and electroactive interferents easily interfere with the amperometric measurement of glutamate [51]. To simulate an environment of glutamate in blood, 200 μM of glutamate solution (150–300 μM of glutamate in whole blood) is used to characterize the selectivity and anti-interference ability of the carbon foam-based sensing system. The interference experiment was carried out by the successive addition of 200 μM of glutamate and a high concentration of 50 μM of different interferent species, including 3,4-Dihydroxyphenylacetic acid (DA), ascorbic acid (AA), uric acid (UA), glucose, and dopamine hydrochloride (DH). The current in Figure 5c showed desirable stability under the addition of interferent species. In addition, the current densities obtained at 200 μM and 400 μM of glutamate are 6.84 mA/cm^2^ and 9.80 mA/cm^2^ (Figure 5c), exhibiting deviations of 6% and 4% from the theoretical values calculated from the equation in Figure 5b, respectively. It indicates satisfactory consistency and repeatability of our glutamate-sensing system. Furthermore, these results demonstrate that the carbon foam-based glutamate sensor possesses satisfactory anti-interference ability and selectivity.

In addition, the stability of our non-enzymatic glutamate detection system was also evaluated from the CA performance for three different electrodes with a relative standard deviation (RSD) of 3.2% in 10 mM PBS containing 4 mM CuCl_2_ and 100 μM of glutamate over one month. The carbon foam-based electrode was stored at room temperature and tested every five days. The current response toward 100 μM of glutamate retained 98.7% of the initial value after 30 days, as shown in Figure 5d. Hence, our non-enzymatic glutamate sensor exhibited impressive stability, which could be essential for glutamate-sensing applications.

## 4. Conclusions

In summary, we developed a novel Cu ion chelation-assisted non-enzymatic glutamate detection system on the porous chitosan-derived carbon foam electrode, which was pyrolyzed from a chitosan foam fabricated through a temperature-controlled freeze-drying process. The porous morphology of the electrode provided a large electroactive surface area, which was 12 times larger than the commonly used gold plate electrode, bringing into a low limit of detection (0.001 μM), a broad detection rate of 10^6^ μM scale (0.001 to 1000 μM) and a high sensitivity of up to 1.9 × 10^4^ μA/mM∙cm^2^. The sensing mechanism of the Cu ions chelation-assisted system was finely investigated and proved to be on account of the redox reactions of the chelation compounds of Cu ions and glutamate. Excellent selectivity was also found for glutamate sensing upon various interferent reagents and the sensing performance of our glutamate sensor retains up to 98.7% after 30 days of regular use. We believe our developed non-enzymatic detection system can achieve a low-cost, facile, sensitive, and broad-spectrum glutamate sensor and can also offer new insights into the detection of other reagents.

## Figures and Tables

**Table 1:** Comparison of the performance of different sensor platforms for glutamate detection.

Electrodes	Enzyme	Linear Range (μM)	Limit of Detection (μM)	Sensitivities (μA/mM∙cm^2^)	Reference
GlutO_x_/cMWCNT-AuNPs-CHIT/Au	GlutO_x_	5–500	1.6	155	[17]
GlutO_x_/Pt-SWCNT/PAA	GlutO_x_	0.05–1600	0.0046	27.4	[31]
PU/GlutO_x_/MWCNT/PPy/Pt	GlutO_x_	0.3–500	0.3	0.384	[48]
GlutO_x_/APTES/ta-C/Pt	GlutO_x_	10–500	10	2.9	[49]
GlutO_x_/BDD/Pt	GlutO_x_	0.5–50	0.35	24	[50]
Glutamate dehydrogenase/CNT/GCE	Glutamate dehydrogenase	2–225	2	0.71	[51]
GlutO_x_/ta-C/CNFs	GlutO_x_	20–500	0.000767	18.8	[12]
GlutO_x_/ZnO nanorods/PPy/PGE	GlutO_x_	0.02–500	0.18	N/A	[52]
GlutO_x_/CeO_2_/TiO_2_/CHIT/o-PD/Pt	GlutO_x_	5–90	0.594	793 pA/µM	[53]
Pt/Ni nanowire array	No enzyme	500–8000	135	65	[16]
NiO/chit/GCE	No enzyme	1000–8000	272	11	[18]
Ni@NC/GCE	No enzyme	0.005–500	135	-	[20]
GluBP/Au NP/SPCE	No enzyme	0.1–0.8	0.15	-	[21]
ZnO/RuO_2_ NPs/GCE	No enzyme	0.0001–10	9.6 × 10^−5^	5.42 × 10^3^	[22]
MWCNT/Ti-doped ZnO/GCE	No enzyme	100–1000 1000–10,000	11.59	25 4.7	[23]
Cu^2+^ assisted carbon foam	No enzyme	0.001–1000	0.001	1.9 × 10^4^	This work

## Data Availability

The data presented in this study are available on request from the corresponding author.

## Supplementary Materials

The following are available online at https://www.mdpi.com/article/10.3390/nano12121987/s1, Figure S1: Scheme of the synthesis process of chitosan-derived graphitic carbon foams, Figure S2: XPS wide scan spectrum (a) and deconvoluted spectra of N 1 s (b) of chitosan foam, Figure S3: (a) Cyclic voltammograms of flat gold electrode for the detection of different concentrations of glutamates (0.5 mM, 1 mM, 1.5 mM, and 2 mM) and carbon electrode for the detection of 1 mM glutamate in 10 mM PBS containing 4 mM CuCl2. The scan rate is 20 mV/s, (b) calibration curve of peak currents of anodic peak in (a) vs. log C, Figure S4: CV curve of the 4 mM CuCl2 aqueous solution measured using the carbon foam electrode, Figure S5: Cyclic voltammograms of carbon foam electrode for the detection of the different concentrations of glutamates (0.5 mM, 1 mM, 1.5 mM, and 2 mM) in 10 mM PBS without CuCl2, Figure S6: (a) Cyclic voltammograms of carbon-based electrode for the detection the different concentrations of glutamates in 10 mM PBS containing 2 mM CuCl2; (b) calibration curve of Ip vs. log C of anodic peak in (a); (c) cyclic voltammograms of carbon-based electrode for the detection of the different concentrations of glutamates in 10 mM PBS containing 6 mM CuCl2; (d) calibration curve of Ip vs. log C of anodic peak 1 in (c), Table S1: EIS data collected from the carbon foam electrode and gold electrode: R_et_, C, and S_A_ represent the electron-transfer resistance, the double-layer capacitance, and the surface area, respectively.
