# Supplementary material for: Porous Carbon Boosted Non-Enzymatic Glutamate Detection with Ultra-High Sensitivity in Broad Range Using Cu Ions
Source: Nanomaterials (Basel). 2022 Jun 9;12(12):1987. doi: 10.3390/nano12121987 (PMC9230436; doi:10.3390/nano12121987)
Supplement: Supplementary file 1 [file nanomaterials-12-01987-s001.zip › nanomaterials-1720295-supplementary.pdf]

nanomaterials

Supplementary Materials

Porous Carbon Boosted Non-Enzymatic Glutamate Detection with Ultra-High Sensitivity in Broad Range Using Cu Ions

Yifei Ma [1], Jiemin Han [1], Zhaomin Tong [1], Jieling Qin [2,*], Mei Wang [1,*], Jonghwan Suhr [3,4], Jaedo Nam [3], Liantuan Xiao [1], Suotang Jia [1] and Xuyuan Chen [1,5]

[1] State Key Laboratory of Quantum Optics and Quantum Optics Devices, Institute of Laser Spectroscopy, Collaborative Innovation Center of Extreme Optics, Shanxi University, Taiyuan 030006, China; mayifei@sxu.edu.cn (Y.M.); jiemin.han@foxmail.com (J.H.); zhaomin.tong@sxu.edu.cn (Z.T.); xlt@sxu.edu.cn (L.X.); tjia@sxu.edu.cn (S.J.); xuyuan.chen@usn.no (X.C.)
[2] Tongji University Cancer Center, Shanghai Tenth People's Hospital of Tongji University, School of Medicine, Tongji University, Shanghai 200092, China
[3] Department of Polymer Science and Engineering, Sungkyunkwan University, Suwon 16419, Korea; suhr@skku.edu (J.S.); jdnam@skku.edu (J.N.)
[4] School of Mechanical Engineering, Sungkyunkwan University, Suwon 16419, Korea
[5] Department of Micro- and Nanosystem Technology, Faculty of Technology and Maritime Sciences, University College of Southeast Norway, 3184 Borre, Norway
* Correspondence: qinjieling770@hotmail.com (J.Q.); wangmei@sxu.edu.cn (M.W.)

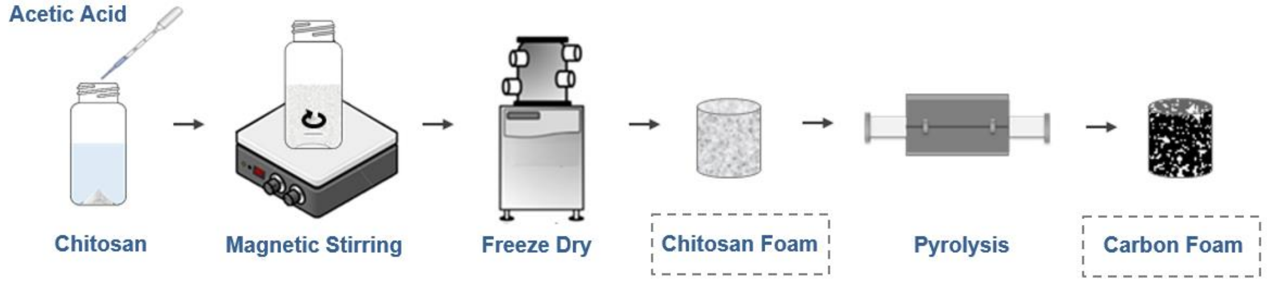

Figure S1. Scheme of the synthesis process of chitosan-derived graphitic carbon foams.

Table S1. EIS data collected from the carbon foam electrode and gold electrode: R_{et}, C, and S_A represent the electron-transfer resistance, the double layer capacitance, and the surface area, respectively.

Electrode	R_{et} (Ω/cm²)	C(×10⁻⁶ F/g)	S_A (cm²/g)
Carbon electrode	0.003388	233.1	11.66
Gold electrode	3.463	19.5	0.98

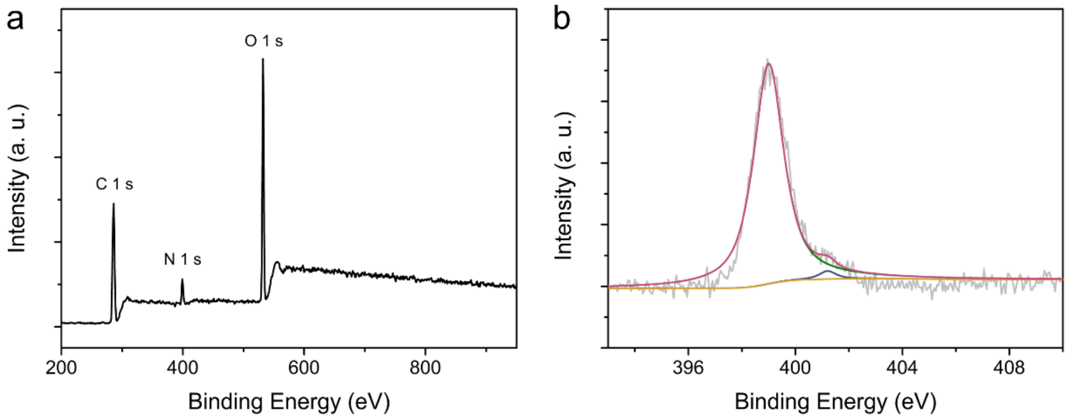

Figure S2. XPS wide scan spectrum (**a**) and deconvoluted spectra of N 1 s (**b**) of chitosan foam.

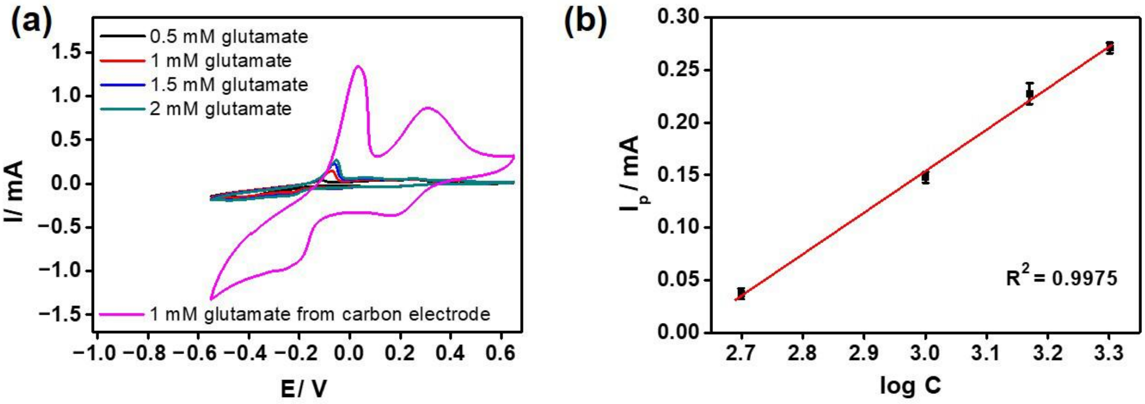

Figure S3. (**a**) Cyclic voltammograms of flat gold electrode for the detection of different concentrations of glutamates (0.5 mM, 1 mM, 1.5 mM, and 2 mM) and carbon electrode for the detection of 1 mM glutamate in 10 mM PBS containing 4 mM $CuCl_2$. The scan rate is 20 mV/s; (**b**) Calibration curve of peak currents of anodic peak in (**a**) vs. log C.

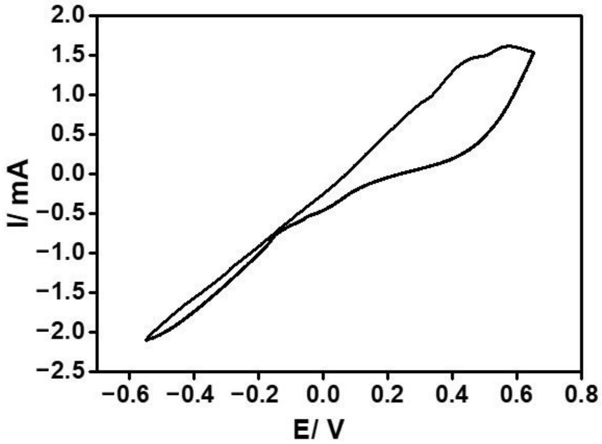

Figure S4. CV curve of the 4 mM $CuCl_2$ aqueous solution measured using the carbon foam electrode.

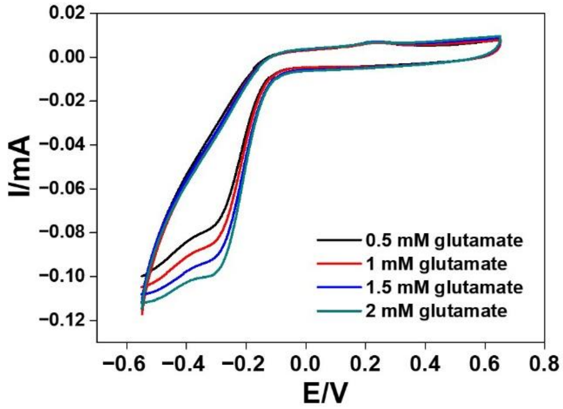

Figure S5. Cyclic voltammograms of carbon foam electrode for the detection the different concentrations of glutamates (0.5 mM, 1 mM, 1.5 mM and 2 mM) in 10 mM PBS without $CuCl_2$.

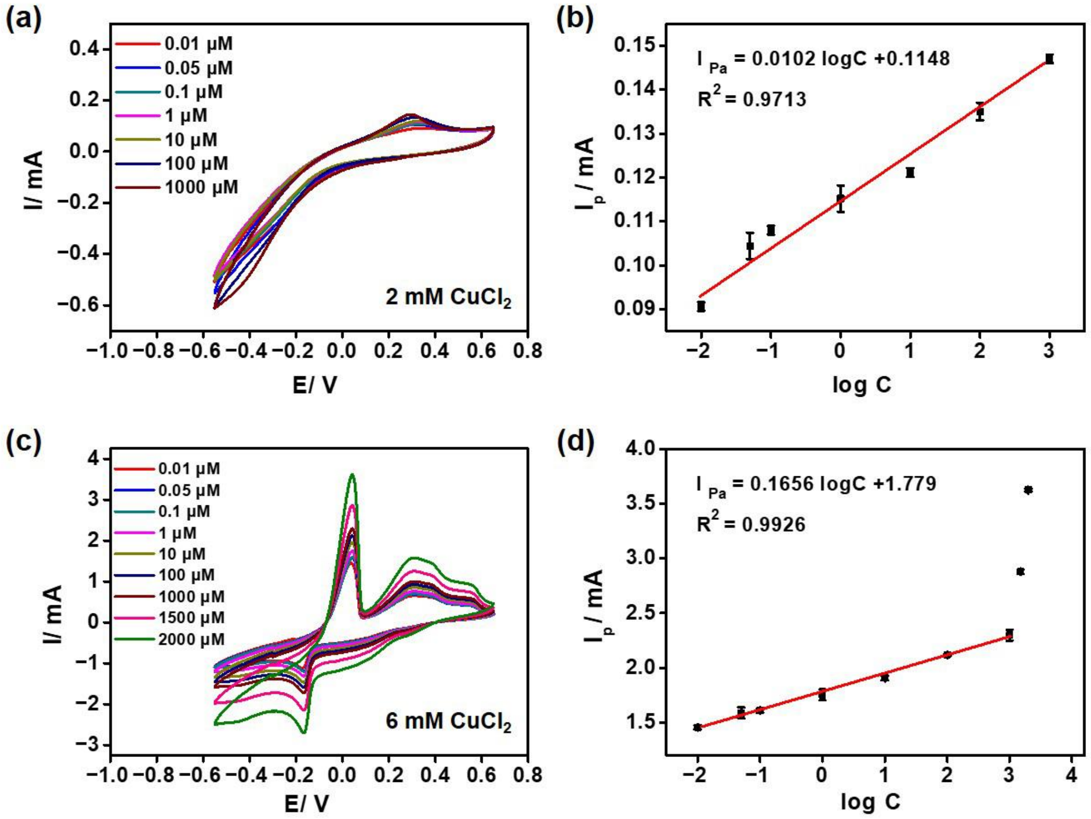

Figure S6. (**a**) Cyclic voltammograms of carbon based electrode for the detection the different concentrations of glutamates in 10 mM PBS containing 2 mM $CuCl_2$; (**b**) Calibration curve of I_P vs. log C of anodic peak in (**a**); (**c**), Cyclic voltammograms of carbon based electrode for the detection the different concentrations of glutamates in 10 mM PBS containing 6 mM $CuCl_2$; (**d**) Calibration curve of I_P vs. log C of anodic peak 1 in (**c**).
